# Depletion of Paraspeckle Protein 1 Enhances Methyl Methanesulfonate-Induced Apoptosis through Mitotic Catastrophe

**DOI:** 10.1371/journal.pone.0146952

**Published:** 2016-01-19

**Authors:** Xiangjing Gao, Guanglin Zhang, Shigang Shan, Yunlong Shang, Linfeng Chi, Hongjuan Li, Yifei Cao, Xinqiang Zhu, Meibian Zhang, Jun Yang

**Affiliations:** 1 Zhejiang Provincial Center for Disease Control and Prevention, Hangzhou, Zhejiang, 310051, China; 2 Collaborative Innovation Center for Diagnosis and Treatment of Infectious Diseases, State Key Laboratory for Diagnosis and Treatment of Infectious Diseases, The First Affiliated Hospital, Zhejiang University, Hangzhou, Zhejiang, 310003, China; 3 Zhejiang CONBA Pharmaceutical Co. Ltd., Hangzhou, Zhejiang, 310058, China; 4 The Affiliated Stomatology Hospital, Zhejiang University School of Medicine, Hangzhou, Zhejiang, 310003, China; 5 Department of Toxicology, Hangzhou Normal University School of Medicine, Hangzhou, Zhejiang, 310016, China; German Cancer Research Center, GERMANY

## Abstract

Previously, we have shown that paraspeckle protein 1 (PSPC1), a protein component of paraspeckles that was involved in cisplatin-induced DNA damage response (DDR), probably functions at the G1/S checkpoint. In the current study, we further examined the role of PSPC1 in another DNA-damaging agent, methyl methanesulfonate (MMS)-induced DDR, in particular, focusing on MMS-induced apoptosis in HeLa cells. First, it was found that MMS treatment induced the expression of PSPC1. While MMS treatment alone can induce apoptosis, depletion of PSPC1 expression using siRNA significantly increased the level of apoptosis following MMS exposure. In contrast, overexpressing PSPC1 decreased the number of apoptotic cells. Interestingly, morphological observation revealed that many of the MMS-treated PSPC1-knockdown cells contained two or more nuclei, indicating the occurrence of mitotic catastrophe. Cell cycle analysis further showed that depletion of PSPC1 caused more cells entering the G2/M phase, a prerequisite of mitosis catastrophe. On the other hand, over-expressing PSPC1 led to more cells accumulating in the G1/S phase. Taken together, these observations suggest an important role for PSPC1 in MMS-induced DDR, and in particular, depletion of PSPC1 can enhance MMS-induced apoptosis through mitotic catastrophe.

## Introduction

Mitotic catastrophe was first described in *Schizosaccharomyces pombe* as a temperature-sensitive lethal phenotype that was observed in some mutant strains and associated with gross abnormalities of chromosome segregation [1, 2]. Similarly, mammalian cell ‘mitotic catastrophe’ had been described as the failure to undergo complete mitosis after DNA damage (coupled to defective checkpoints). After several cell cycles, this situation would lead to tetraploidy or endopolyploidy with extensive DNA damage, perhaps followed by the selection of apoptosis-resistant cells that would ultimately survive after endo reduplication [3, 4]. Nowadays, the term ‘mitotic catastrophe’ is used to define a specific type of cell death occurring during mitosis or resulted from failed mitosis[5, 6]. Usually, when the mitotic apparatus is damaged, cell cycle checkpoints will be activated and arrest cells in the G2/M phase, thereby preventing a cell from entering mitosis with damaged or under-repaired DNA. However, failure to arrest these cells at or before mitosis results in the formation of multinucleated giant cells that contain abnormal nuclei, which is one of the most prominent morphological characteristics of cells undergoing mitotic catastrophe [7]. This process is associated with senescence and considered to be a major cause of DNA damage-induced cell death [8]. Thus, mitotic catastrophe is regarded as a special case of apoptosis [9, 10].

Methyl methanesulfonate (MMS) is a typical methylating agent that has been used as a model experimental research chemical, as well as a solvent catalyst in polymerization, alkylation, and esterification reactions [11]. MMS can serve as an alkylating agent that causes single point mutations[12]. MMS is also a known genotoxic compound that can directly react with guanine and adenine bases of DNA to generate interstrand and intrastrand cross-links [13]. MMS can stall replication forks at the sites of DNA cross-links in dividing cells, resulting in the formation of DNA double-strand breaks, which are regarded as one of the most detrimental forms of DNA damage [14, 15]. Double-strand breaks can activate several signal transduction pathways including DNA repair, cell cycle checkpoints, mitotic catastrophe and apoptosis [16].

Paraspeckle protein 1 (PSPC1) was first identified as a structural protein present in a specific type of nuclear body called the paraspeckle [17]. To date there were only limited studies regarding the functions of PSPC1, and thus its overall functions have not been comprehensively characterized. Still, it had been reported that PSPC1 could be phosphorylated by the serine/threonine protein kinases ataxia telangiectasia mutated (ATM) and ataxia telangiectasia and Rad3-related protein (ATR), key mediators of the cellular DNA damage response (DDR) [18]. We had also shown that PSPC1 could be induced by cisplatin treatment in a proteome study [19]. Based on such observations, we further conducted a series of experiments in an effort to decipher the possible function of PSPC1 in cisplatin-induced DDR. As it turned out, PSPC1 is important for cisplatin-induced G1/S arrest, since knock-down of PSPC1 could abrogate such arrest and lead cells entering the G2/M phase [20]. Furthermore, a significant increase of apoptotic cells was also observed in PSPC1 knock-down cells [20]. Taken together, these data indicate the important function of PSPC1 in cisplatin-induced DDR, and specifically, as a regulator of the G1/S checkpoint.

However, besides the cisplatin-induced DDR, whether PSPC1 is involved in other chemical-induced DDR is not clear. In addition, although knock-down of PSPC1 resulted in increased cell death, the underlying molecular mechanism remained unclear. Therefore, in the current study, we analyzed the role of PSPC1 in MMS-induced DDR, and in particular, focusing on its effects on MMS-induced apoptosis. As reported here, we have shown that PSPC1 is also involved in MMS-induced DDR, and specifically, PSPC1 depletion enhanced MMS-induced apoptosis through mitotic catastrophe.

## Material and Methods

### Materials

Human cervical carcinoma (HeLa) cells were purchased from ATCC(Manassas, VA). Minimal essential medium (MEM) and fetal bovine serum (FBS) were purchased from GibcoInvitrogen Corp. (Gibco Laboratories, Grand Island, NY). 3-(4,5-Dimethylthiazol-2-yl)-2,5-diphenyl-2H-tetrazoliumbromide (MTT), methyl methanesulfonate (MMS) and 4ʹ,6-diamidino-2-phenylindole (DAPI) were purchased from Sigma-Aldrich (St. Louis, MO). The Annexin V-fluorescein isothiocyanate (FITC)/propidiumiodide (PI) apoptosis detection kit and β-actin antibody were obtained from MultiSciences Biotechnology (Hangzhou,China). FITC-conjugated anti-mouse secondary antibody was obtained from Zhongshan Biotechnology (Beijing, China). γH2AX antibody was supplied by Millipore (Billerica, MA); and an affinity-purified peptide antibody against PSPC1 was generated in rabbits in our laboratory as described by Fox *et al*[21]. Alexa Fluor 488-conjugated goat anti-mouse and goat anti-rabbit IgG were obtained from Life Technologies (Carlsbad, CA).

### Cell culture and cell cycle synchronization

HeLa cells were grown in MEM supplemented with 10% FBS with 5% CO_2_ at 37°C. Cell cycle synchronization was carried out by double thymidine blockage at the G1/S boundary, as described before[22]. Briefly, cells were grown in the presence of 2 mM thymidine (Sigma, St. Louis, MO) for 18 h, then washed with phosphate-buffered saline (PBS), and grown in fresh medium without thymidine for 8 h. Thymidine was added again at 2 mM and incubated for a further 18 h to block cells at the G1/S boundary.

### Assessment of cell viability

Cell viability was determined by the mitochondrial-dependent reduction of MTT ((3-(4,5-dimethylthiazol-2-yl)-2,5-diphenyltetra-zolium bromide) as previously described [23]. In short, cells were grown in 96-well plates (5×10^3^cells/well) and treated with the indicated concentration of MMS. At 12, 24, 36 and 48 h after treatment, the supernatant was removed and 100 μL (500μg/mL) MTT solution was added to each well. After 4 h of incubation at 37°C, the reaction solution was carefully aspirated and then 150 μL DMSO was added into each well for 10 min to dissolve the formazan crystals. The reduction of MTT was quantified by measuring the absorbance at 570 nm using the Multi-Detection Microplate Reader (Synergy 2, Bio-Tek Instrument Inc, USA). Data were expressed as mean ± SD from six parallel wells in 3 repeated experiments.

### Immunofluorescence microscopy

For immunofluorescent staining, cells were fixed in 4% paraformaldehyde for 15 min, permeabilized with 0.5% triton, and blocked with 3% bovine serum albumin for 1 h at 37°C. The cells were incubated with anti-γH2AX primary antibodies overnight, washed three times in PBS, and then incubated with Alexa Fluor 488-conjugated secondary antibodies for 1 h. DNA was counterstained with 1 mg/mL DAPI for 15 min at 37°C. Cells mounted on coverslips were observed with a Leica DMI 4000 immunofluorescent microscope or a Zeiss confocal laser scanning microscope.

### Immunoblotting

Cells were lysed in RIPA lysis buffer (Beyotime, Nantong, China), and protein concentrations were determined using the bicinchoninic acid Protein Assay Kit (Beyotime). Denatured protein extracts were loaded and separated on 15% or 8% sodium dodecyl sulfate-polyacrylamide gels (Mini-Protean II, Bio-Rad) and transferred to a polyvinylidene fluoride membrane (Millipore). After blocking with 3% non-fat milk in Tris-buffed saline with 0.1% (v/v) Tween-20, membranes were incubated with primary antibodies at 4°C overnight, followed by incubation of Alexa Fluor 488-conjugated secondary antibodies for 1 h at room temperature. After three washes, membrane-bound proteins of interest were detected using an Odyssey Infrared Imaging System (Li-Cor, USA).

### Transfection of small interfering RNA (siRNA) and detection of PSPC1 expression

Two sets of siRNA oligonucleotides for the human PSPC1 gene, corresponding to nucleotides 1257–1275 (siPSPC1), and a negative control siRNA were synthesized by Shanghai GenePharma Co., Ltd. siRNAs were transfected into HeLa cells using Lipofectamine2000 (Invitrogen, Carlsbad, CA), as described by the manufacturer and using a siRNA concentration of 40 nM. In short, cells were seeded into a 6-well cell culture plate, siRNA-Lipofectamine2000 complexes were added to each well after 24 h, and the medium was changed after a 6-h incubation. After 24 h, protein levels were determined by Western immunoblotting.

### Plasmid vectors and transfection

The plasmid for PSPC1 overexpression (pPSPC1) and the negative control plasmid (pCON) were constructed by Shanghai Genechem Co., Ltd.. Cells were transfected with 2 mg plasmid or empty vector in Opti-MEM medium (Invitrogen) using X-treme GENE HP DNA transfection reagent (Roche), following the manufacturer’s protocol.

### Analysis of apoptosis

The Annexin V-FITC/PI kit (Multiscience) was used to analyze the extent of apoptosis. Briefly, cells were collected by trypsinization, washed three times with PBS, and then resuspended in 500 μL binding buffer with 5 mL Annexin V-FITC and 10 μL PI. Cells were incubated for 5 min in the dark at room temperature. The cells were then analyzed using a FC500 MCL machine (Beckman Coulter) at 10,000 events/sample.

### Cell cycle analysis

For flow cytometry measurements of the cell cycle, 36 h-post transfection cells were trypsinized, centrifuged at 300 X *g* for 5 min, and fixed overnight in 70% cold ethanol at -20°C. After washing twice with PBS, the cells were resuspended in 500 μL of fresh PBS containing 50 μL of 2 mg/mL RNaseA and 10 μL of 1 mg/mL PI (Sigma). Cells were incubated for 15 min at 37°C. The cells were then analyzed immediately using a FC500 MCL machine (Beckman Coulter) at10,000 events/sample.

### Statistical analysis

Statistical analyses were performed using Student’s t-test or one-way analysis of variance. Each experiment was conducted at least three times independently. Data were presented as mean ± the standard deviation (SD) and a probability level of P<0.05 was considered statistically significant.

## Results

### Effect of MMS on cell viability and DNA damage

**Fig 1:**
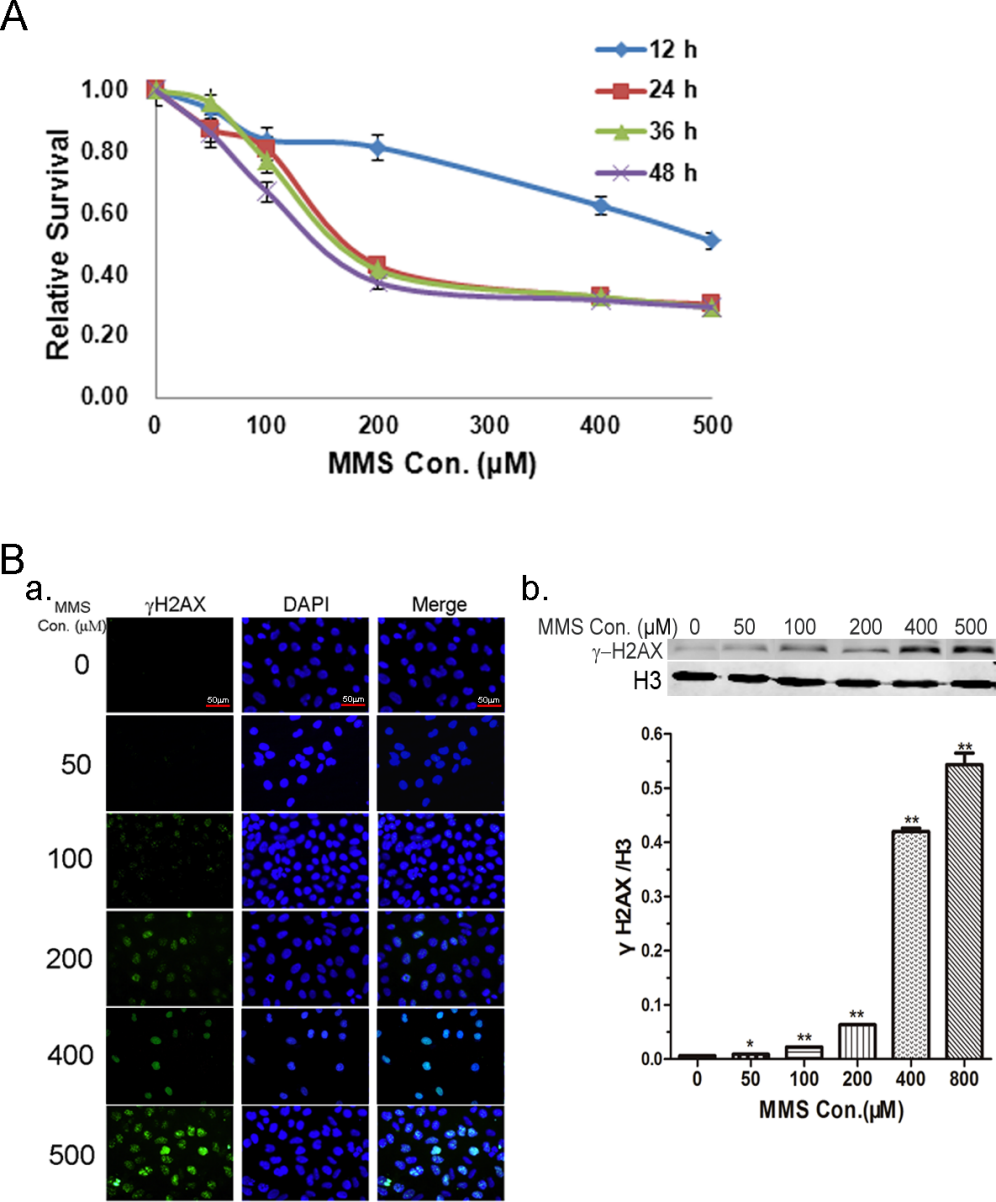
Cell viability and DNA damage were determined after methyl methanesulfonate (MMS) treatment. (A) HeLa cells were treated with the indicated concentrations of MMS for 12, 24, 36, and 48 h. Cell viability was measured by MTT assay. (B) HeLa cells were treated with different concentrations of MMS for 12 h, then detected the level of DNA damage by immunofluorescence (a) and Western blot (b). Scale bar, 50 μm. The results are shown as the mean ± SD of three independent experiments. *p < 0.05, **p < 0.01, as compared with the solvent control group.

The cytotoxic effects of different concentrations (0, 50, 100, 200, 400, and 500 μM) of MMS on HeLa cells were assessed using the MTT reduction assay after 12, 24, 36, and 48 h of treatment. As shown in Fig 1A, cell viability showed no marked change at 12 h for 50, 100 and 200 μM of MMS employed. Also, there was no significant change in viability in the presence of 50μM for any of the time-periods investigated (P > 0.05). However, HeLa cell viability was significantly decreased by exposure to 200, 400, and 500 μM MMS for 24, 36, and 48 h (P < 0.01).

Based on the MTT assay results, we treated cells with 0, 50, 100, 200, or 400 μM MMS for 12 h, and then investigated MMS-induced DNA damage by assessing the γH2AX protein level using immunofluorescence and Western blotting. Shown in Fig 1B were representative immunofluorescence and immunoblot images of the γH2AX protein. It was found that while there was only a very low level of γH2AX in untreated cells, exposure to 50, 100, 200,or 400 μM MMS caused a concentration-dependent increase in the γH2AX level. This observation indicated that MMS can significantly induce DNA damage in HeLa cells.

### PSPC1 is induced by MMS

**Fig 2:**
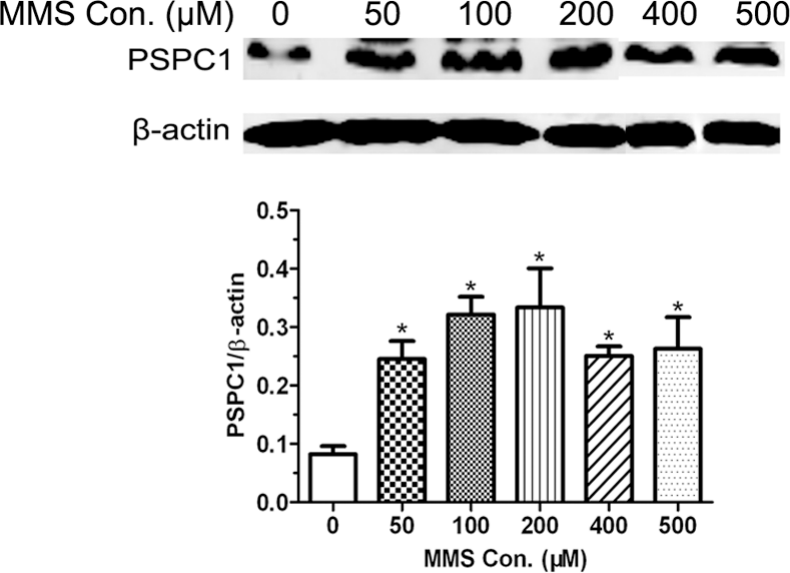
Paraspeckle protein 1 (PSPC1) is induced by MMS. HeLa cells were treated with indicated concentrations of MMS for 12 h. The levels of PSPC1 were examined by Western blot. The results are shown as the mean ± SD of three independent experiments. *p < 0.05, as compared with the control group.

Previously we have demonstrated that the DNA-damaging agent cisplatin could induce PSPC1 expression in HeLa cells [20]. To determine whether MMS also had such effect, HeLa cells were treated with different concentrations of MMS for 12 h, and the expression of PSPC1 was examined by Western blot. As shown in Fig 2, the level of PSPC1 is indeed increased in a concentration-dependent manner by exposure to MMS at a concentration of under 200 μM. The PSPC1 level showed a small reduction, though still much higher than control, at MMS concentrations above 200 μM, which could be the result of less cells due to the significant decline in cell viability (Fig 1A).

### Modulation of PSPC1 influences MMS-induced cell cycle redistribution

**Fig 3:**
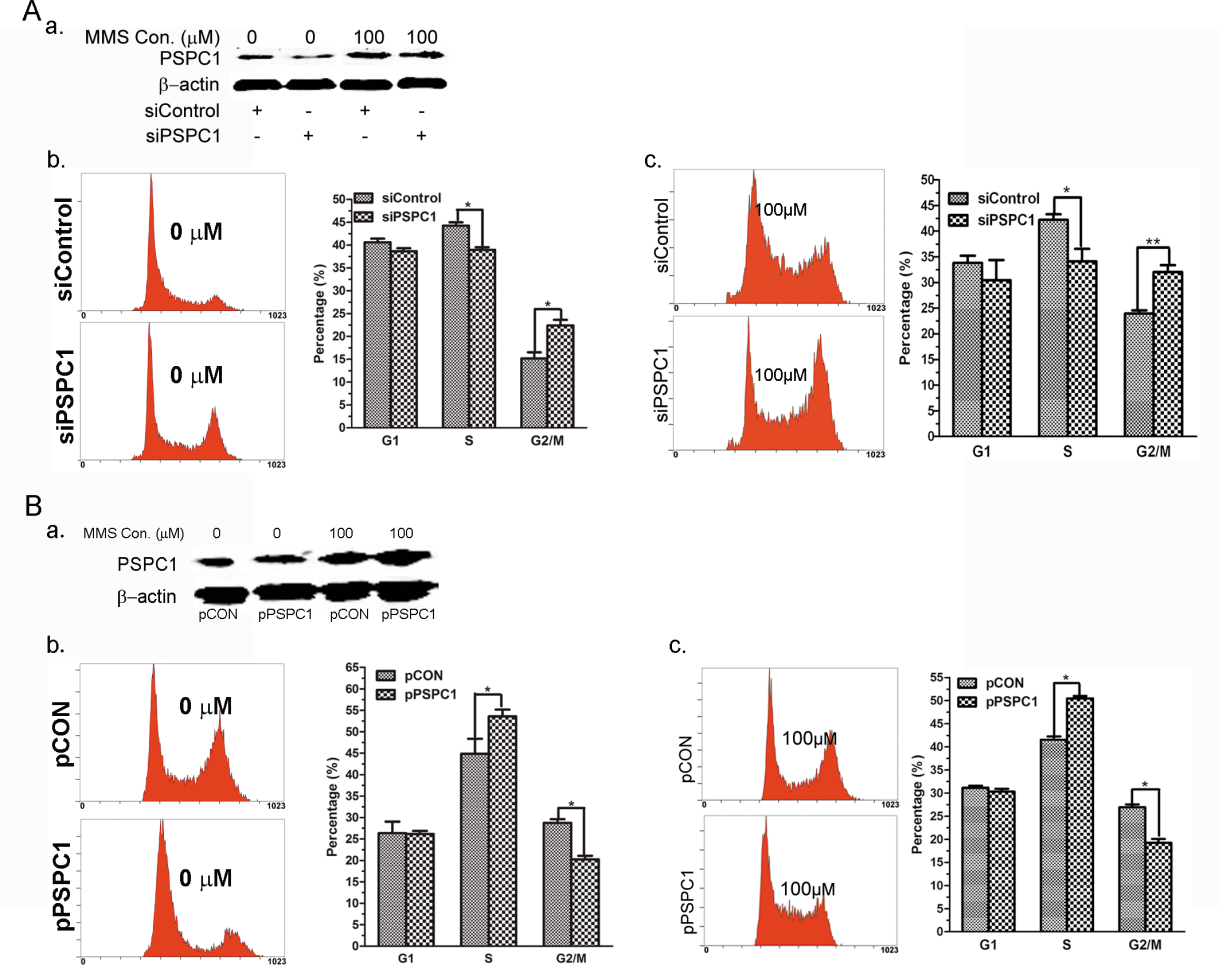
Modulation of PSPC1 influences MMS-induced cell cycle redistribution. (A) HeLa cells were transfected with siPSPC1 or siControl for 24 h, then treated with 0 μM and 100 μM of MMS for 12 h. The expression of PSPC1 was examined by Western blot and cell cycle was analyzed by flow cytometry. (B) HeLa cells were transfected with pPSPC1 or pCON to overexpress PSPC1 for 24 h, then treated with 0 μM and 100 μM of MMS for 12 h. The expression of PSPC1 was examined by Western blot and cell cycle was analyzed by flow cytometry. The values represent averages of three independent experiments, mean ± SD.*p < 0.05, **p < 0.01, as compared with the control group.

One of the key functions we identified for PSPC1 was its involvement in cisplatin-induced G1/S arrest, as knock-down of PSPC1 abrogated the G1/S arrest [20]. Here we also used the PSPC1 knock-down model to evaluate its effects on cell cycle distribution. Western blot analyses showed that transfection with PSPC1 siRNAs consistently reduced PSPC1 protein expression by about 95%, even with MMS treatment, as compared with the level observed in cells transfected with control siRNA (Fig 3A and S1 Fig). Similar with our previous results, for siPSPC1 cells, there is a great increase in the number of cells entering G2/M. However, unlike cisplatin, which arrested more than 50% cells in S phase, MMS treatment only caused a mild increase in the percentage of S phase cells (35% in G1, 42% in S, and 23% in G2/M phase after MMS treatment in control siRNA-transfected cells). Nonetheless, knock-down of PSPC1 led to decreased S phase cells and increased G2/M phase cells (30%in G1, 35% in S, and 35% in G2/M) (Fig 3A), which is consistent with the cisplatin results.

To further confirm the function of PSPC1 in G1/S arrest, we established a stable cell line that overexpresses PSPC1 (pPSPC1). Similarly, MMS treatment caused a mild increase in S phase in control plasmid-transfected cells (30% in G1, 40% in S, and 30% in G2/M phase, Fig 3B). Interestingly, for pPSPC1 cells after MMS treatment, the cell cycle distribution was 30% in G1, 50% in S, and 20% in G2/M, representing a marked increase in S phase and decrease in G2/M phase (Fig 3B). Together, these results further strengthen the function of PSPC1 in cell cycle regulation during DDR.

### Effect of PSPC1 on MMS-induced apoptosis

**Fig 4:**
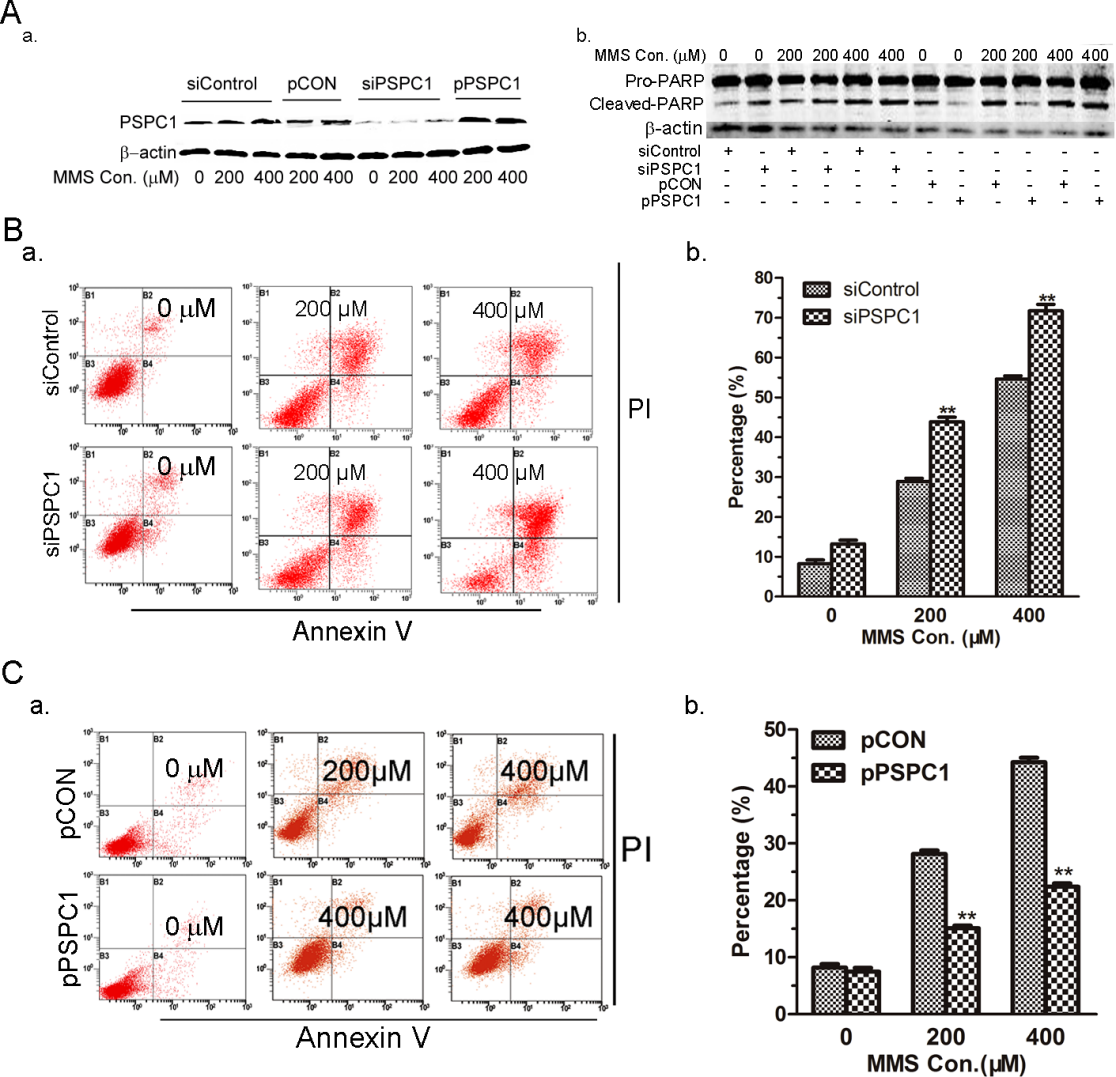
Effect of PSPC1 on MMS-induced apoptosis. (A) HeLa cells were transfected with siPSPC1, siControl, pPSPC1, or pControl for 24 h, followed by 200 or 400 μM of MMS treatment for 12 h prior to analysis of PSPC1 and PARP expression by Western blot. For the detection of apoptosis, HeLa cells transfected with siRNAs (B) or overexpression plasmids (C) were treated with 200 or 400 μM of MMS for 12 h, and then analyzed by dual-parameter flow cytometry utilizing Annexin V-FITC and PI double staining. Representative dot plot data from three independent experiments are shown in the left panel, and the histogram graph in the right panel represents the percentage of dual-parameter positive cells pooled from three independent experiments. Data are presented as the mean ± SD of three independent experiments. **p < 0.01, as compared with the control group.

We then further evaluated the effects of PSPC1 on MMS-induced apoptosis. Unlike cisplatin, MMS induced more pronounced apoptosis in HeLa cells (over 30% compared to 10–15% by cisplatin). Still, similar to what we observed in cisplatin-treated cells, PSPC1-knockdown rendered HeLa cells more sensitive to the toxic effect of MMS, and more cells died of apoptosis (over 45% and 70% in 200 and 400 μM MMS treated cells, respectively, Fig 4B). Furthermore, we detected the level of cleaved PARP to confirm the effect of PSPC1 in MMS-induced apoptosis. To further verify that PSPC1 expression indeed influenced MMS-induced apoptosis, pPSPC1 cells were subjected to MMS treatment. As shown in Fig 4C, overexpression of PSPC1 in HeLa cells significantly inhibited MMS-induced apoptosis, which was only half of the respective controls. Together, these findings suggested that PSPC1 can influence MMS-induced apoptosis.

### Knockdown of PSPC1 induces mitotic catastrophe

**Fig 5:**
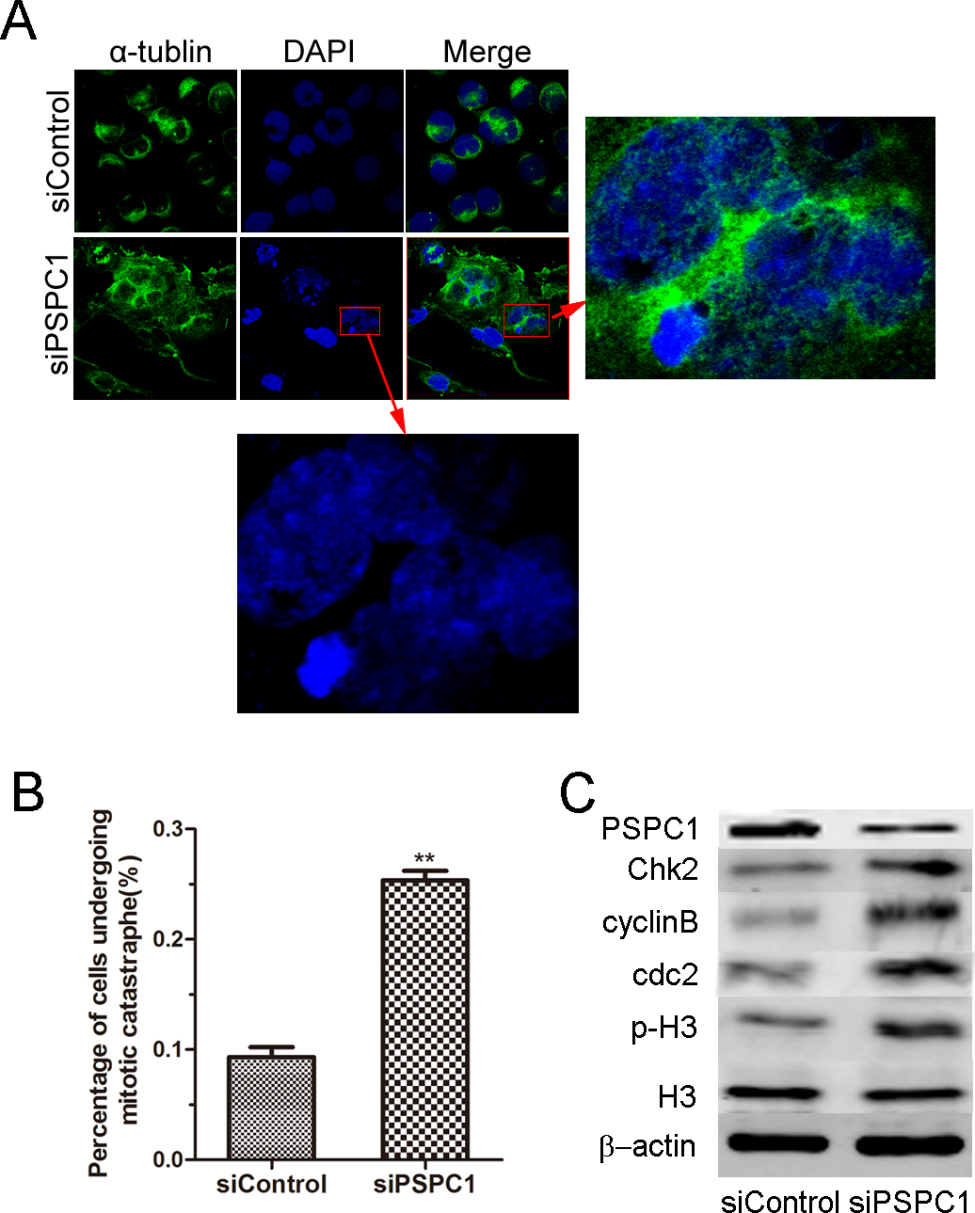
Knockdown of PSPC1 induces mitotic catastrophe. HeLa cells were transfected with siPSPC1 or siControl. 24 h post-transfection, cells were harvested and the expression of tubulin and the nucleolus were examined by immunofluorescence microscopy (A). The percentage of multinuclear cells were shown as a histogram, and densitometry data of three independent experiments were presented, mean ± SD (n = 200 cells). (B). (C) Using the same treated cells, the levels of representative mitotic catastrophe proteins (phospho-histone H3 [Ser10], Cdc2, cyclinB, and Chk2) were examined by western blot. **p < 0.01, as compared with the control group.

To answer how PSPC1 depletion could enhance apoptosis, we first examined cell morphology using light microscopy after knockdown of PSPC1. Interestingly, it was found that after MMS treatment, many of the PSPC1-knockdown cells failed to separate completely, resulting in the formation of some “giant”cells. Furthermore, the nuclei of such cells were not homogonously stained, showing the presence of multiple “micronuclei”, which was indicative of the occurrence of mitotic catastrophe (Fig 5A). Then we counted the number of such abnormal cells in control and siPSPC1 groups, and as shown in Fig 5B, PSPC1 knockdown induced a marked increase in cells undergoing mitotic catastrophe. To confirm whether these cells were indeed undergoing mitotic catastrophe, the expression levels of phospho-histone H3, cdc2, cyclinB, and Chk2, known markers of mitotic catastrophe [24–27], were measured by Western blot. As shown in Fig 5C, PSPC1 knockdown markedly increased the level of phospho-histone H3, as well as the levels of cdc2, cyclinB, and Chk2. Therefore, these data pointed out that the depletion of PSPC1 can lead to mitotic catastrophe, thus explaining the increased cell death up on MMS-induced DNA damage.

## Discussion

PSPC1 was first found in paraspeckles in transcriptionally active cells and in perinucleolar caps of cells that were not actively transcribing Pol II genes[21]. Previously, most of the known functions of PSPC1 had been related to regulate either gene expression or RNA processing [21, 28], for example, PSPC1 had been found to be involved in the regulation of mRNA splicing and androgen receptor-mediated transcriptional activity [29]. However, based on ours and other groups’ observation [18, 19], it was hypothesized that PSPC1 might also have important functions in DDR. A further detailed study revealed that PSPC1 did participate in the DNA-damaging agent cisplatin-induced DDR, and in particular, it acted in the G1/S checkpoint, as knockdown of PSPC1 resulted in failed G1/S arrest [20].

In the current study, we further expand the investigation to other DNA-damaging agent, in an effort to confirm what we have found in cisplatin-induced DDR. Therefore, MMS, a well-known alkylating agent, was employed. Even though the two chemicals have distinct DNA-damaging properties, e.g., alkylating vs cross-linking, similar responses were observed for PSPC1. First, PSPC1 can be induced by MMS (Fig 2). Second, PSPC1 is also involved in MMS-induced G1/S arrest. This is supported by the observation that knockdown of PSPC1 led cells escape MMS-induced G1/S arrest (Fig 3B). In contrast, overexpressing PSPC1 caused a more pronounced G1/S arrest (Fig 3C). And finally, depletion of PSPC1 by siRNA enhanced MMS-induced apoptosis, while overexpressing PSPC1 decreased the number of cells undergoing apoptosis (Fig 4). Such observation is consistent with other groups’ results, which also examined the effects of knocking down PSPC1 on apoptosis under DNA damage conditions [30, 31]. Taken together, these data reinforced the conclusion that we have drawn from the cisplatin study, pointing out the important function of PSPC1 in DDR, especially in the G1/S regulation.

G1/S arrest is a key process of DDR, during which time allows the cells to repair the various types of DNA damage [32]. Many molecules are involved in the G1/S arrest machinery, including CDKs, cyclins, Chk1, Chk2, ATM, ATR and so on. The key checkpoint response to DNA damage in G1/S is the ATM (ATR)/Chk2 (Chkl) induced stabilization and activation of p53 [33]. There are several possible mechanisms for a specific molecule to regulate G1/S arrest. For example, the sensor kinases ATM/ATR can phosphorylate and activate the effector kinases Chk2/Chk1, which in turn phosphorylate Cdc25, and then Cdc25 activates cyclin E-CDK2 complex to arrest cell cycle[34, 35]. Still, upon DNA damage, the tumor suppressor protein p53 is increased, and then initiates the transcription of downstream genes, including p21, which in turn activates the G1/S arrest [36]. On the other hand, retinoblastoma protein (pRB) uses a different mechanism, for example, protein-protein interaction—pRB prevents premature entrance into S phase by binding to a number of cellular proteins such as the transcription factors E2F-1-5 which regulate the expression of S phase genes [34]. What specific mechanism PSPC1 utilizes to regulate the G1/S arrest currently is not clear. Still, as PSPC1 is known to regulate gene expression, it is possible that upon DNA damage, the increased PSPC1 protein can activate the expression of certain genes that are involved in G1/S arrest. However, since PSPC1 itself has not been classified as a transcription factor, its function in regulating gene expression could be the result of working together with some other transcription factors, or at the post-transcription level, such as mRNA processing, or even translation. Nonetheless, it is also possible that PSPC1 could interact directly with certain molecules of the G1/S checkpoint machinery. These possibilities are currently under investigation in our laboratory.

The other interesting aspect of PSPC1 is its relationship to apoptosis. Our results showed that depletion of PSPC1 significantly increased apoptosis, while overexpression of PSPC1 produced a dramatic decrease in the number of apoptotic cells following MMS treatment (Fig 4). This phenomenon is a characteristic of proteins participating in apoptosis; for example, altered caspase 3 expression is related to the degree of apoptosis [37]. However, it should be kept in mind that such observation did not necessarily indicate PSPC1 as a component of the apoptosis machinery, but more like, as a regulator of apoptosis.

Furthermore, our immunofluorescence results showed that in PSPC1-depleted cells, there were many giant cells with altered nuclear morphology (Fig 5), which is the most prominent characteristic of mitotic catastrophe. Therefore, the expression levels of some key proteins which are involved in mitotic catastrophe were examined, and the results showed that they were upregulated, further supporting the observation of aberrant mitosis (Fig 5C). In addition, it is generally believed that cells undergoing mitotic catastrophe would have a 4N DNA content, which indicates the G2/M phase of cell cycle. In consistent with this notion, cell cycle analysis results showed that in PSPC1 knockdown cells, the percentage of cells in the G2/M phase was significantly increased. These observations confirmed the involvement of PSPC1 in mitotic catastrophe.

It is well-known that mitotic catastrophe is a process that leads to apoptotic cell death [8]. This process is widely believed to occur in cells with unrepaired DNA damage; and when these cells enter mitosis prematurely, they cannot complete cell division and this eventually leads to cell death [38–40]. Considering our results showing that (i) knockdown of PSPC1 was associated with increased apoptosis; and (ii) loss of PSPC1 increased the number of cells showing mitotic catastrophe, it is reasonable to deduce that PSPC1 knockdown induces mitotic catastrophe, leading to increased apoptosis. Similarly, polo-like kinase 1 (Plk1), which is involved in mitotic arrest, has also been reported to induce mitotic alteration and apoptosis [10, 41–43].

In conclusion, our study demonstrated that attenuation of PSPC1 expression influences MMS-induced DDR, and in particular, depleting PSPC1 can enhance MMS induced apoptosis through mitotic catastrophe. Combined with our previous cisplatin study, these findings provide novel insights into the functions of PSPC1, specifically for its function in DDR. Further study is required to reveal the detailed molecular mechanisms underlying PSPC1-induced cell death, as well as the exact mechanisms of PSPC1 in other aspects of the DDR.

## Supporting Information

S1 Fig Knockdown of PSPC1 influences MMS-induced cell cycle redistribution. HeLa cells were transfected with 2^nd^ set of siPSPC1 or siControl for 24 h, then treated with 0 μM and 100 μM of MMS for 12 h. The expression of PSPC1 was examined by Western blot (A) and analyzed by flow cytometry (B).
(TIF)
