# Supplementary figures and images for: Depletion of Paraspeckle Protein 1 Enhances Methyl Methanesulfonate-Induced Apoptosis through Mitotic Catastrophe
Source: PLoS One. 2016 Jan 19;11(1):e0146952. doi: 10.1371/journal.pone.0146952 (PMC4718682; doi:10.1371/journal.pone.0146952)

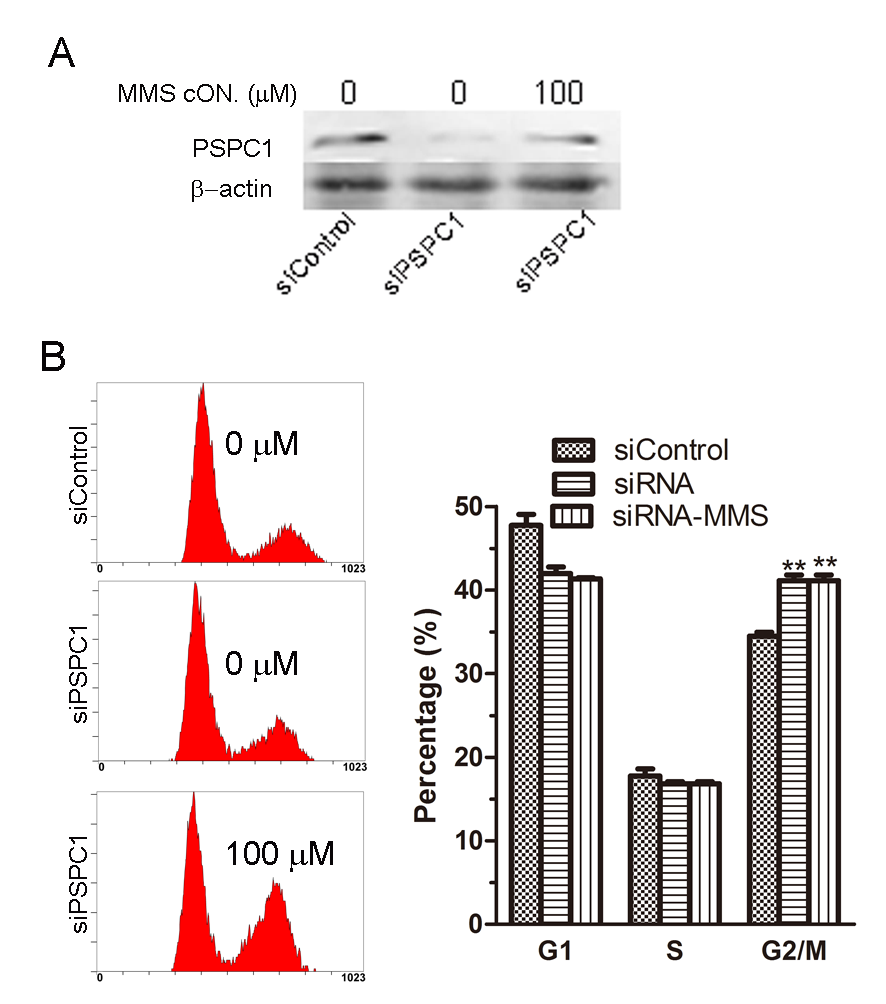

Supplement: S1 Fig — HeLa cells were transfected with 2nd set of siPSPC1 or siControl for 24 h, then treated with 0 μM and 100 μM of MMS for 12 h. The expression of PSPC1 was examined by Western blot (A) and analyzed by flow cytometry (B). (TIF) [file pone.0146952.s001.tif]
